# Magnetic Resonance Texture Analysis in Myocardial Infarction

**DOI:** 10.3389/fcvm.2021.724271

**Published:** 2021-10-28

**Authors:** Fei Peng, Tian Zheng, Xiaoping Tang, Qiao Liu, Zijing Sun, Zhaofeng Feng, Heng Zhao, Lianggeng Gong

**Affiliations:** ^1^Department of Medical Imaging Center, Second Affiliated Hospital of Nanchang University, Nanchang, China; ^2^Department of Radiology, The First Affiliated Hospital, Hengyang Medical School, University of South China, Hengyang, China

**Keywords:** magnetic resonance imaging, myocardial infarction, texture analysis (TA), machine learning, stratifying risk

## Abstract

Texture analysis (TA) is a newly arisen field that can detect the invisible MRI signal changes among image pixels. Myocardial infarction (MI) is cardiomyocyte necrosis caused by myocardial ischemia and hypoxia, becoming the primary cause of death and disability worldwide. In recent years, various TA studies have been performed in patients with MI and show a good clinical application prospect. This review briefly presents the main pathogenesis and pathophysiology of MI, introduces the overview and workflow of TA, and summarizes multiple magnetic resonance TA (MRTA) clinical applications in MI. We also discuss the facing challenges currently for clinical utilization and propose the prospect.

## Introduction

Myocardial infarction (MI) is myocardial necrosis caused by ischemia and hypoxia of cardiomyocytes, an imbalance between oxygen offering and myocardial requirement. It belongs to a part of the clinical manifestation of the acute coronary syndrome (ACS) (1). MI is a major cause of death and disability worldwide and brings about approximately one-third of all deaths in patients over 35 years old (2, 3).

The European Society of Cardiology (ESC) has conducted the fourth universal definition of MI from cardiac troponin values (cTn) and clinical myocardial ischemia evidence that is ranging from symptoms of myocardial ischemia, ECG abnormalities, and new imaging evidence (4). However, clinical ischemic symptoms are not specific for myocardial ischemia and may be misdiagnosed as other medical conditions (5). Early mortality and morbidity can be decreased by accurate diagnosis, better prevention, and management, then life expectancy and quality of life will be enhanced (6). So, more sensitive, precise, and specific techniques are required for the diagnosis and characterization of MI.

As the imaging techniques evolve, it plays a more and more important role in MI. Cardiac magnetic resonance (CMR) has advantages, such as non-Radiative, multiparameters and sequences, multiplanar reconstruction capabilities, and high tissue resolution, which composes the “gold standard” tool for evaluating the cardiac structure and function non-invasively and becomes the best available imaging technique for detection of MI (7, 8). Meanwhile, CMR has essential significance in stratifying risk, predicting prognosis, predicting response to therapy, detecting complications, etc., in MI (8). Nevertheless, the traditional visual inspection of images may not recognize subtle differences and detect invisible signal changes (9, 10). Magnetic resonance texture analysis (MRTA), which belongs to radiomics, includes extensive technologies modeling the spatial distribution of pixel grayscale for data recognition, classification, and segmentation based on the latent texture. Furthermore, MRTA is capable of detecting subtle signal changes and obtaining underlying image information that remains imperceptible to eyes to provide tissue characteristics (9, 11–13). More importantly, MRTA has the potential further to strengthen the diagnostic and prognostic value of imaging (14, 15).

This article concentrates on the role of MRTA in MI. In this study, we review the major pathogenesis and pathophysiology of MI, the basic concepts and types of MRTA, the clinical applications of MRTA in MI, current challenges, and potential prospects.

## Pathogenesis and Pathophysiology of MI

Myocardial infarction is most commonly owing to coronary thrombosis from the rupture of an atherosclerotic plaque (16). When the blood is exposed to the thrombogenic lipids, the platelets and coagulation factors are activated, which become the precipitating factor of the plaque disruption (3). The coronary plaques with lipid-rich core and thin fibrous cap occupy the highest risk of rupture (17). However, in addition to atherosclerosis, there are several etiologies of MI, such as intracardiac thrombus or valvular vegetation that led to coronary artery embolism, cocaine use, coronary dissection, hypotension, anemia, Kawasaki's syndrome, trauma, metabolic disease, congenital coronary anomalies, and complications of angiography (3, 17).

In MI, once the onset of myocardial ischemia, there is a subsequent decrease in myocardial perfusion, bringing about the reduction of tissue oxygenation, which transforms the hypoxia cardiomyocytes from aerobic to anaerobic metabolism, bringing about edema of cardiomyocyte and ultimately tissue necrosis (5).

**Figure 1:**
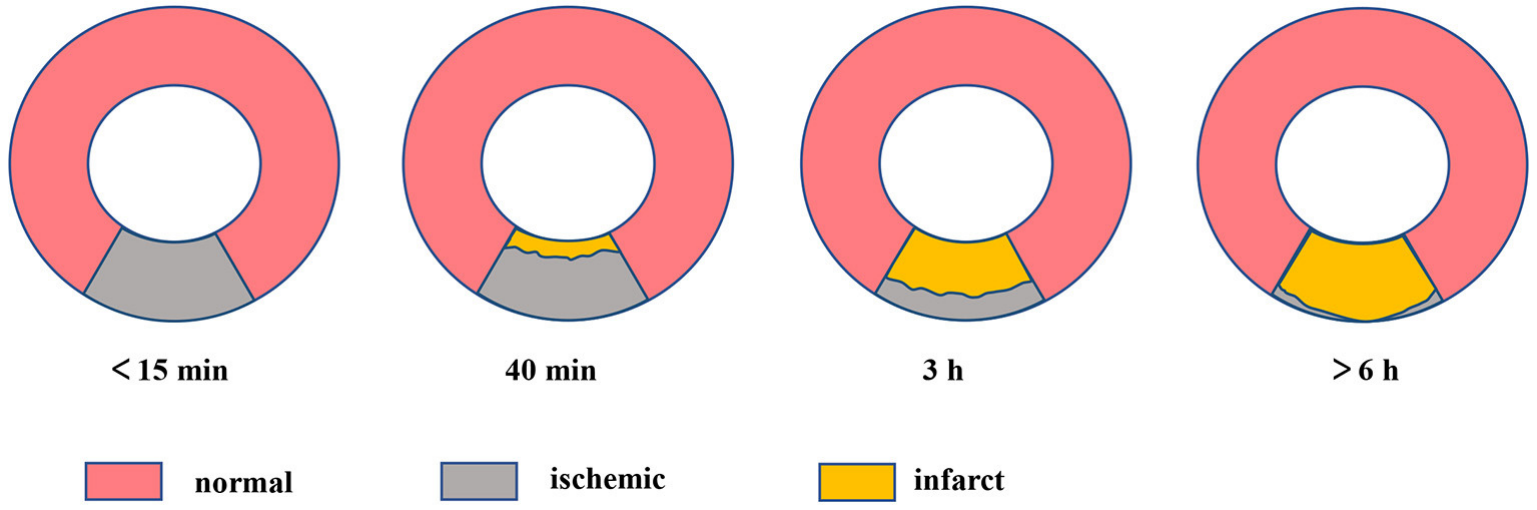
The “wavefront” of myocardial ischemic necrosis, necrosis of cardiomyocytes progresses from the subendocardium to the subepicardium over time if ischemia persists and ultimately becoming transmural infarct.

The myocardial necrosis process resembles a “wavefront” phenomenon that is ranging from the endocardium to the epicardium (18) (Figure 1). Within about 15 min, the myocardium shows ischemia with no infarct. At approximately 40 min, it appears subendocardial infarction. Roughly at 3 h, the subendocardial infarct extends to the mid myocardium. Beyond 6 h, the infarction extends to subepicardial layers and develops into transmural. After approximately 2 months, scar tissues replace the piece, inflammatory cells, and edema, leading to shrinking of the necrotic tissue and myocardial thinning (5, 19).

## Texture Analysis

**Figure 2:**
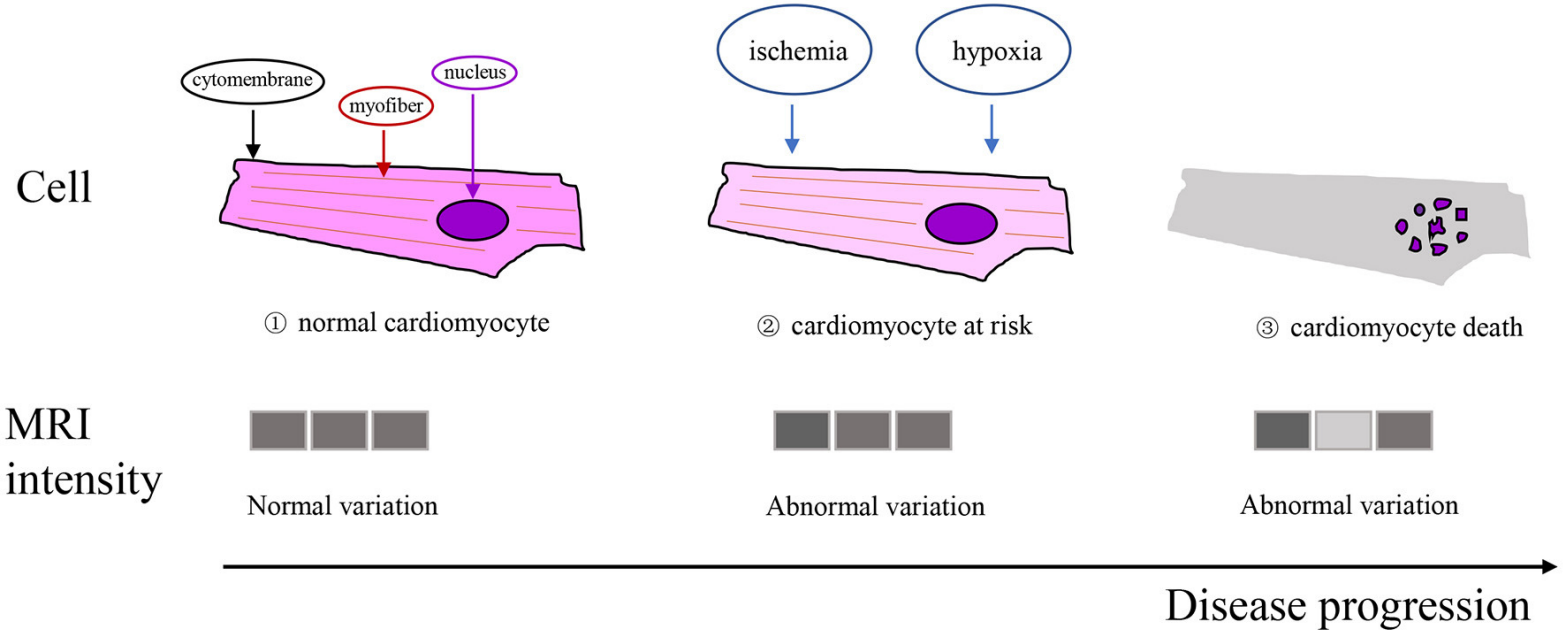
Schematic illustration of the texture working hypothesis in myocardial infarction (MI). Top row: ischemia and hypoxia may cause cardiomyocyte at risk or death; (1), (2), and (3) represent normal cardiomyocyte, cardiomyocyte at risk, and cardiomyocyte death, respectively. Bottom row: changes in the statistical properties of the image intensities due to the persistent ischemia and hypoxia of cardiomyocytes may be reflected as certain textural patterns.

**Figure 3:**
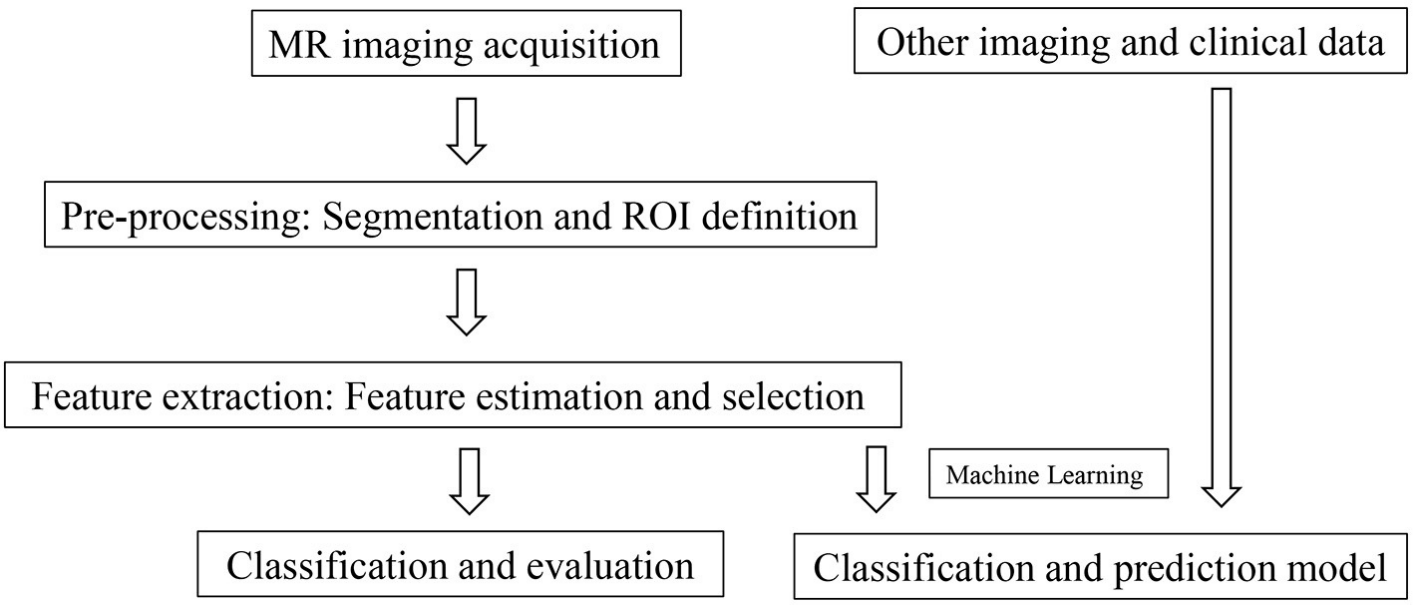
Overview of the MRTA simplified workflow. MRTA, magnetic resonance texture analysis.

**Figure 4:**
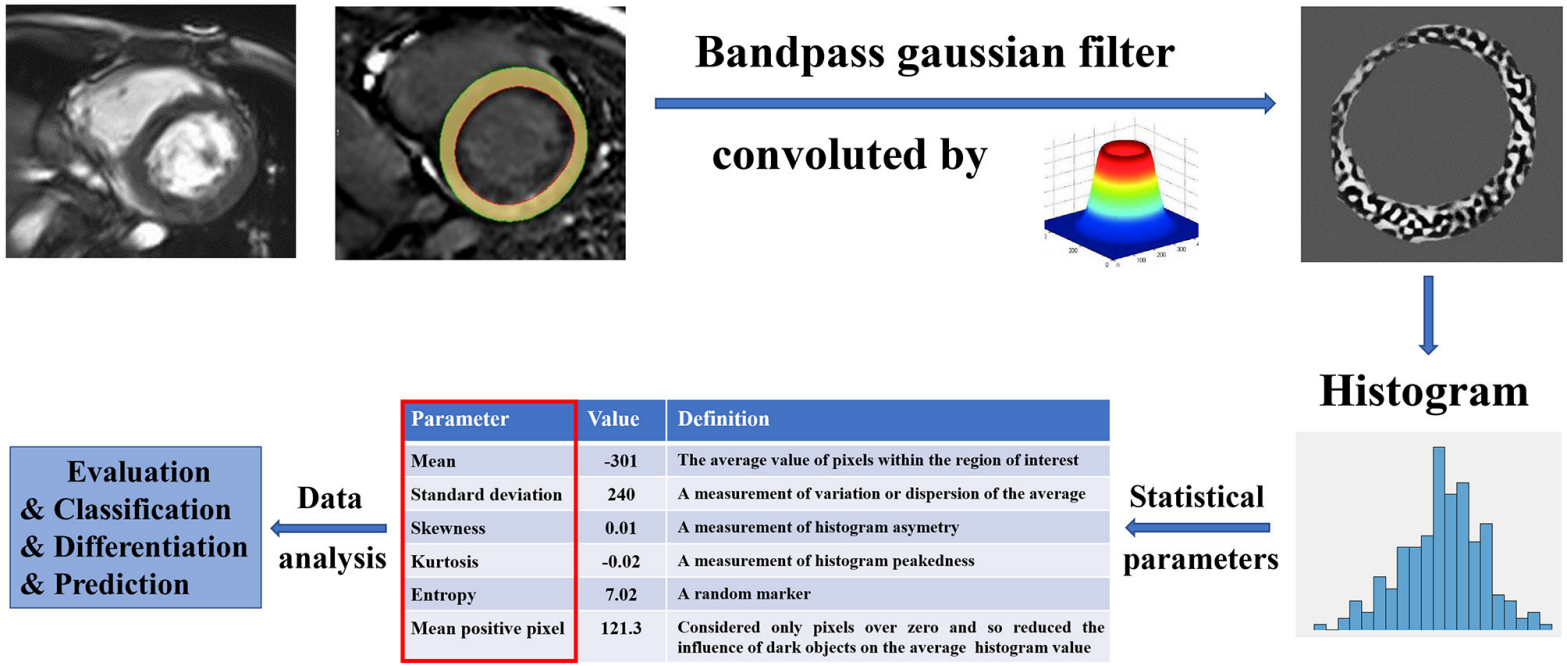
A schematic diagram illustrating the whole texture analysis (TA) applied on CMR. The myocardium in a CMR image was convolved by a bandpass Gaussian filter to enhance the image and highlight the variance. The histogram of the enhanced myocardium was computed to obtain a couple of statistic parameters, such as mean, SD, skewness, kurtosis, entropy, and mean positive pixel. These parameters were furthermore analyzed among different patients for evaluation, classification, differentiation, and prediction. CMR, Cardiac magnetic resonance.

Texture analysis (TA) is a part of radiomics. Through image post-Processing technology, the distribution and relationship of pixel or voxel gray are analyzed to extract many quantitative texture features that are not visible by the naked eyes in medical images (20). The image texture represents the gray-level variation rule of pixels in images (21). Changes in image intensity owing to persistent ischemia and hypoxia may be reflected as textural patterns near or after cardiomyocyte death (Figure 2). Figure 3 exhibits a simplified workflow about the clinical application of MRTA. A schematic diagram illustrating the whole TA applied on CMR is shown in Figure 4. In the light of the means applied to assess the inter-relationships of the pixels, the forms of texture analyses can be classified as below: statistical, structural, model-based, and transform methods (13, 14).

### Types of TA

With high gray and spatial resolution, MRI images possess extensive and similar image information. Therefore, the statistical method becomes the most commonly used method in MRTA. For statistical-based TA, various properties control the distribution and relations of gray-scale values in images to represent texture (14). First-order statistical TA, also called a histogram, extracts the image intensity values from the region of interest (ROI). A histogram can be derived by calculating the frequency count of the number of pixels of each gray value (22). Second-order statistics analyze the spatial relationship or co-occurrence of the pixel intensity values. The two most commonly used methods are gray-level co-occurrence matrix (GLCM) and gray-level run-length matrix (GLRLM). Through the calculation of the neighborhood gray difference matrix, high-order statistics study the spatial relationship among three or more pixels and reflect the change of intensity in a specific area or the distribution of homogeneous areas. The common calculation method includes neighborhood gray-tone difference matrix (NGTDM) and gray-level size zone matrix (GLSZM) (23).

Structural-based TA catches intensity changes between the central and adjacent voxels. Local binary patterns (LBP) are a non-Parametric algorithm and also the most frequently used method that depicts the local features of gray-scale connection between image pixels and neighboring pixels (24).

Model-based TA explicates texture in an image with sophisticated mathematical models, for example, stochastic or fractal models. The model parameters are estimated and applied to image analysis. Due to a lack of direction selectivity, this method is unsuited for depicting local image structures (13).

Transform-based TA enables the spatial information of images to convert into spatial frequencies. It contains Gabor, Fourier, and Wavelet transform. As the most widespread method, Wavelet transform can analyze the frequency content of images in different spatial frequency resolutions (25).

### Machine Learning

Artificial intelligence (AI) is rapidly gaining importance in the medical imaging field and is likely to gradually turn into clinical practice in the next few years (26, 27). According to the data derived from AI, machine learning is a rapidly growing area that concentrates on building systems that make accurate predictions according to the data (28). Machine learning is mainly applied to establish the diagnosis of MI and assists differential diagnosis of acute mesenteric ischemia (AMI) and chronic mesenteric ischemia (CMI) that cannot be identified by the naked eyes (15, 29, 30). The application of machine learning in medical imaging can be briefly summarized into three types: supervised, unsupervised, and semisupervised learning (31). Supervised learning intends to recognize the relationship between characteristics relevant to the learning objectives and the expected result indicators in a dataset for classification. Unsupervised learning intends to identify and establish potential patterns through the use of unlabeled data from a computer. By combining supervised and unsupervised learning, semisupervised learning utilizes the amount of unlabeled data and few labeled data for training. At present, the most relevant to cardiovascular imaging are supervised and unsupervised learning (32).

## MRTA Clinical Applications in MI

Magnetic resonance texture analysis clinical applications in MI emerge as a promising research domain, and many findings have presented encouraging results. The series of studies can be divided into three broad categories: establishing the diagnosis, determining the age of infarct, stratifying risk (Table 1).

**Table 1:** Magnetic resonance texture analysis (MRTA) clinical applications in myocardial infarction (MI).

**Clinical application**	**References**	**Study size**	**Acquisition**	**Type of TA**	**Modeling methods**	**Results**
Establishing diagnosis	Baessler et al. (15)	120	CMR, 1.5T	Histogram, GLCM, GLRLM, absolute gradient, wavelet autoregressive model,	Multiple logistic regression	Combining the texture features Teta1 and Perc.01 obtain the highest accuracy for diagnosing large and small MI on CMR with an area under the curve of 0.93 and 0.92 respectively
Determining age of infarct	Larroza et al. (29)	44	LGE, CMR, 1.5T	Histogram, GLCM, GLRLM, absolute gradient, wavelet autoregressive model,	Random forest algorithm SVM	The polynomial SVM yielded the best classification performance (AUC = 0.86 ± 0.06 on LGE and AUC = 0.82 ± 0.06 on cine MRI)
	Chen et al. (33)	70	T1 mapping, LGE, CMR, 3.0T	Histogram, GLCMDifference entropy,GLRLM	Random forest algorithm	GLRLM features (horizontal fraction) extracted from of ECV demonstrated a significantly higher AUC (0.91) than other texture features in differentiation of unsalvageable infarction and salvageable myocardium
Risk stratification	Larroza et al. (34)	50	Cine, CMR,1.5T	GLCM, GLRLM, GLSZM, NGTDM, LBP	SVM	Evaluation of myocardial segmental viability based on transmural extension: LBP features using a 2D + t approach achieved high discrimination (AUC > 0.8) of non-Viable, viable and remote segments, with sensitivity 92, 72, and 85%, respectively
	Ma et al. (35)	68	T1 mapping, CMR, 3.0 T	Histogram, GLCM, wavelet	Multiple logistic regression	The combination of TA and T1 mapping native T1 values could provide high diagnostic accuracy for transmurality (AUC = 0.84) and MVO (AUC = 0.86)
	Kotu et al. (36)	22	LGE, CMR, 1.5T	Dictionary-based texture,dc-values	MLE-Bayes	TA aided with intensity values gives better segmentation of scar from myocardium with high sensitivity (82.32%) and specificity (89.05%)
	Kotu et al. (37)	44	LGE, CMR,1.5T	Dictionary-based texture	FTCM	Pixel with larger Rp values is more likely to be the border area of scar
	Engan et al. (38)	24	LGE, CMR	GLCM	MLE-Bayes	Combination of image texture and statistical features on scar may have potential discriminative power between high and low risk of serious ventricular arrhythmias groups
	Gibbs et al. (39)	76	LGE, CMR, 1.5 T	Histogram	Kaplan-Meier analysis	Patients suffering arrhythmic events with significantly higher kurtosis and lower skewness compared with those suffering no arrhythmic events

*CMR, cardiac magnetic resonance; LGE, late gadolinium enhancement; GLCM, gray-level co-occurrence matrix; GLRLM, gray-level run-length matrix; GLSZM, gray-level size zone matrix; NGTDM, neighborhood gray-tone difference matrix; SVM, support vector machine; MLE, maximum likelihood estimator; LBP, local binary patterns; ECV, extracellular volume; MVO, microvascular obstruction; AUC, area under the curve; TA, texture analysis; FTCM, Frame texture classification method*.

### Establishing Diagnosis

Despite the relative importance of various MRI technologies vary, MRI plays a significant role in establishing a diagnosis for both AMI and CMI (7). The rapid application of TA in medical imaging provides a new method for diagnosing MI. Baessler et al. (15) performed TA in 120 patients with MI using histogram, GLCM, GLRLM, absolute gradient, autoregressive model, and wavelet transform. Taking late gadolinium enhancement (LGE) as a reference standard, five texture features [Teta1, WavEnHH.s-3, Perc.01, S (5,5) Sum Entrp, and Variance] enabled distinguishing scarred myocardium from normal myocardium on non-Enhanced cine MRI independently. Moreover, multiple logistic regression showed that Teta1 and Perc.01 achieved the highest diagnostic performance for small and sizeable myocardial scars with the area under the curve (AUC) were 0.92 and 0.93, respectively. Therefore, MRTA may provide an extra mean for the diagnosis of MI with gadolinium-free enhancement imaging, which may be helpful for patient groups with accompanying chronic kidney disease who have an added risk of gadolinium-related complications.

### Determining the Age of Infarct

Apart from being a useful diagnostic tool, MRI can also be applied for differentiation between acute and chronic infarction, which is especially helpful when the patient has multi-infarct in different vascular areas or when an infarct occurs with no clinical symptoms. Distinguishing AMI from CMI has vital clinical significance for treatment and follow-up, especially in patients with pre-Existing CMI, and the probability of locating acute lesions by ECG or coronary angiography is limited. The management of two types of infarcts differs. It is crucial to determine the infarct age, especially when both infarction entities coexist, complicating that will complicate the therapeutic plan and follow-up after treatment. Several imaging features involving the identification of AMI and CMI in previous studies, such as wall thickening and thinning (40), microvascular obstruction (MVO) (41), edema on T2-weighted images (42, 43), and hyper-enhancement in contrast-enhanced MRI (44). However, some of these imaging features lack insufficient sensitivity and specificity, and technical limitations still exist (7, 29).

Compared with those current technologies that rely on image visual evaluation, the quantitative character of TA is a unique advantage. Edema of AMI and fibrosis of CMI hold the most essential characteristics of cardiac pathological changes correspondingly and affect the internal structure of tissues, which indicates there may be some inherent texture discrepancy in the images of tissues influenced by AMI and CMI. Larroza et al. (29) performed TA in 44 patients with MI (22 with AMI and 22 with CMI) by using histogram, GLCM, GLRLM, absolute gradient, autoregressive model, and wavelet transform, 279 texture features extracted from cine, and LGE MRI were used to distinguish AMI from CMI alone. A nested cross-validation approach combining a feature selection technology called multiple support vector machine recursive feature elimination (MSVM-RFE) was applied to test the diagnosis efficiency, the results showed that the polynomial SVM achieved the optimum classification performance (AUC = 0.86 ± 0.06 on LGE MRI and AUC = 0.82 ± 0.06 on cine MRI). However, the discrimination is not straightforward and demands the use of machine learning, especially in cases where the infarction is not readily visually perceptible in standard cine MRI.

### Stratifying Risk

In recent years, MRTA plays a new role in identifying multiple prognostic indicators that guide risk stratification and prognosis prediction in patients with MI. The applications mainly involve five sub-aspects: (1) differentiation of reversible from irreversible myocardial injury; (2) evaluation of transmurality; (3) detection of MVO; (4) assessment of scar size combined with segmentation; and (5) identification of scar heterogeneity.

#### Differentiation of Reversible From Irreversible Injury

Differentiating reversible from irreversible myocardial damage is valuable for prognosis prediction. The salvaged myocardium is an independent predictor of prognosis and is associated with mortality, representing the risk area but can be rescued or rescued through revascularization (45). The myocardial salvage index (formula: T2-weighted edema area-delayed enhanced area/T2-weighted edema area) (45), T1 mapping techniques (46, 47), blood oxygen-level dependent (BOLD) (48), etc., have been studied to evaluate the severity of the ischemic myocardial injury. Nevertheless, the traditional visual quantification of the average intensity level for ROI is insufficient because it easily ignores subtle changes and remains subjective (49). TA may overcome some of the above limitations by utilizing specific imaging information to obtain tissue features and quantitatively analyze the relationship between pixels and gray patterns in images.

One promising result about the differentiation of reversible from irreversible myocardial injury based on MRTA was reported by Bing-Hua Chen et al. (33). They examined TA of extracellular volume (ECV) mapping from the calculation of native and post-Contrast T1 mapping images in 70 patients with ST-elevation MI. Five texture features [one co-occurrence matrix features S (0,1) difference entropy, two histogram indexes (mean and perci.99%), and run-length matrix features (horizontal fraction and vertical fraction)] were selected for analysis. The results showed that the horizontal fraction demonstrated a significantly higher AUC (0.91) than other texture features in identifying unsalvageable and salvageable myocardium.

#### Evaluation of Transmurality

The transmurality of infarction possesses an independent prognostic value in measuring the recovery of contractile function after treatment, and a greater transmural extent is related to poorer recovery (50, 51).

Magnetic resonance texture analysis shows several promising results in the evaluation of transmurality. Ma et al. (35) combined T1 mapping and TA for the assessment of myocardial segmental transmurality in 68 patients with AMI, the combination of native T1 values and four features {histogram (mean), GLCM [S(0,1) Correlat, S(1,-1) SumEntrp, and S(2,0) Correlat]} achieved good diagnostic performance (AUC = 0.84). In addition, taking the transmural extension on LGE as the standard judgment of infarcted segmental viability, Larroza et al. (34) applied MRTA to distinguish infarcted viable, non-Viable, and remote segments in 50 patients suffering CMI. Features derived from four matrix-based TA (GLCM, GLRLM, GLSZM, and NGTDM) and LBP methods were extracted from the segments on cine MRI, by combining SVM classifier, LBP using a 2D + t method achieved high discrimination (AUC > 0.8), with sensitivity 92% (non-Viable), 72% (viable), and 85% (remote), respectively. Hence, MRTA may have the potential to provide a new way to detect the myocardial segmental transmurality and assess myocardial segmental viability by the gadolinium-free method.

#### Detection of MVO

Microvascular obstruction appears in severe microcirculation damage caused by myocyte death, overflow of intracellular substances, severe stasis, and occlusion of end arteries and capillaries (7). Moreover, MVO often indicates transmural MI and correlates with adverse remodeling and poor prognosis (52).

Ma et al. (35) applied MRTA for the detection of MVO in 68 patients with AMI. Through combination of native T1 values and eight texture features {[histogram(Perc. 90%), GLCM [S(1,0) Entropy, S(0,1) Correlation, S(4,0) SumVarnc, S(5,0) DifEntrp], and wavelets (WavEnLL_s-1, WavEnLL_s-2, WavEnLL_s-3)]} that are extracted from T1 mapping, it reached a high diagnostic performance (AUC = 0.86) for MVO. Thus, MRTA may provide a more accurate means for diagnosing the severity of the acute myocardial injury.

#### Assessment of Scar Size Combined With Segmentation

The extent of infarct can predict left ventricular adverse remodeling (LVAR) and correlates negatively with prognosis (53–55). Besides that, since the scar is a cause of arrhythmia, infarct size is a better predictor of ventricular tachycardia than left ventricular (LV) ejection fraction (EF) or LV volumes (56, 57). Previous reports suggest the scar size assessed by MRI has the potential to predict survival and mortality independent of LVEF (58–60). Thus, segmentation of scar is a first step to explore the internal information in the scar.

Magnetic resonance texture analysis-combined segmentation can better provide the signal intensity features of the scar, then to help better segment the scar and assess scar size. Kotu et al. (36) segmented scar from normal myocardium on LGE MRI using intensity-based TA in 22 post-MI patients. Through maximum likelihood estimator (MLE)-based Bayes classification, the dictionary-based texture features and dc-values were applied to segment scarred and normal myocardium. Compared with manual segmentation by cardiologists, TA aided with intensity values achieved better segmentation of scar with high sensitivity (82.32%) and specificity (89.05%), thus may be helpful to reduce small missed infarcts that not even affect LVEF but can lead to arrhythmic events (61–63).

#### Identification of Scar Heterogeneity

After MI, the necrotic tissue is gradually replaced by granulation and fibrous tissue and finally, develops into scar tissue. The myocardium presents heterogeneous nature owing to scarring, and the scar tissue is complex on a histopathological level. In addition, numerous studies have shown that the degree of scar heterogeneity correlates directly with the risk of arrhythmia events, thereby, better guide the implantation of an implantable cardioverter defibrillator (ICD) (64, 65). In previous research, the myocardial heterogeneity visualization is mainly based on thresholds that are defined at intensity levels corresponding to the percentage of the max intensity level in scar area (57, 66). TA allows for quantization of patterns and relations among pixels inside images occasionally invisible to human eyes, thus, acquiring an additional measure of heterogeneity.

The scar can be described as two areas: (1) the core area, which consists of fibrous tissue and the myocardial fibers which are in a state of complete death, does not respond to the electrical signals transmitted by the myocardium to tell the heart to contract; (2) the border area, also known as peri-infarct area or gray zone area, contains heterogeneous tissue composed of necrotic tissue mix with bundles of viable cardiomyocytes; the electrical signals in these areas will be disturbed, which may lead to reentry and sometimes arrhythmias (37, 39, 57). It is believed that accurately defining and visualizing border areas may be helpful to give insight into better risk stratification for patients with MI. A probability mapping technique based on texture and intensity features was proposed by Kotu et al. (37) to describe the heterogeneity of myocardial scar in CMR images after MI. On dictionary-based textural features, the result showed that the pixel with larger Rp values was more likely to be the border area of the scar but not necessarily in scar core, which may offer an additional valuable means to identify border areas in scarred myocardium to help predict arrhythmia events.

Some studies reveal that MRTA helps to identify arrhythmias events for patients with previous MI. Engan et al. (38) performed data analysis of image texture (based on GLCM) and statistical features on 24 implanted ICD patients with myocardial scars in CMR. Using maximum likelihood estimation (MLE)-based Bayes classifiers, the results showed that a combination of texture and statistical features might have potential discriminative power between the high and low risk of severe ventricular arrhythmias groups. Similar findings were reported by Gibbs et al. (39) who applied MRTA by using filtration histogram technique to assess LGE images in 76 patients with previous MI and evaluated the characteristics of the scar heterogeneity. The average follow-up time was 371.5 days to observe the ventricular arrhythmic events. The results suggested that patients who are suffering from arrhythmia presented higher kurtosis and lower skewness compared with those suffering no arrhythmia; furthermore, Kaplan-Meier analysis revealed that higher coarse kurtosis and lower fine skewness possessed a particular ability to forecast the increased incidence of ventricular arrhythmic events.

## Discussion

### Challenges of MRTA

Despite these superiorities, extensive clinical application of MRTA in MI remains challenging. First, most of the studies are small sample pilot exploration in a single center, and almost all of them are retrospective studies. The conclusions obtained lack extensive verification support, and the clinical evidence is not sufficient. Therefore, prospective, multicenter, and large sample studies are needed. Second, at present, medical imaging equipment lacks the same image acquisition and imaging algorithm standard, and different acquisition times of the same MR machine and acquisition of different MR machines can affect the stability of radiomics features, and the reproducibility is low. Consequently, strict protocols and standardized methodologies should be followed to maximize the validity of future research. Third, there are many feature parameters in TA, and the prediction accuracy is affected by feature parameters, feature selection methods, and classifiers. In addition, MRTA software is manifold, and there is no evidence to show which software is superior at present. However, 3D TA includes more spatial information and is superior to 2D TA but applies less due to increased complexity. Thus, more accurate and widely applicable feature selection and pattern recognition methods will become the development direction of MRTA.

### Comparison of MRTA and CMR

In the era of big data, TA has become a research hotspot in the field of precision medicine and AI (25). Compared with CMR, MRTA has many advantages as follows. First, MRTA can detect tissue changes that are not easily perceptible to the naked eyes by quantifying gray-level patterns and pixel interrelationships in images, thus can compensate the deficiency of traditional image analysis methods and strengthen the utilization value of CMR images simultaneously (10). Second, MRTA can recognize subtle differences in textural information and further strengthen the diagnostic, prognostic, and predictive values of CMR imaging (67). In addition, MRTA has several shortcomings when compared with CMR, lack of standardization has become the main reason that limits its widespread clinical application (68); besides, TA software needs to be purchased for an additional fee and thus causes increased costs. Although the development of MRTA is still at an early stage and faces many challenges, MRTA shows good clinical application prospects in the cardiovascular field.

### Prospects

Although multiple MRTA clinical applications in MI have shown encouraging results, some aspects are not or rarely referred to and may become future developments.

In traditional imaging, the following factors are also related to risk stratification and prognosis in patients with MI that may be helpful for MRTA clinical application, for instance, (1) hemorrhage in the core of infarct has been demonstrated as an adverse prognostic indicator that is relevant to LVAR, large infarct size, and increased LV end-systolic volume (69); (2) ischemia may help to identify the individuals at high risk of non-Fatal MI; moreover, compared with patients with no peri-infarct ischemia, the existence of peri-infarct ischemia correlates with a higher incidence of cardiovascular events (70); (3) the right ventricle (RV) function evaluated late after MI is also a significant prognostic indicator (71). In the future study, MRTA applications in evaluating hemorrhage in the core of infarct, ischemia, and the RV function, etc., may be of potential value for risk stratification and prognosis prediction in MI. Besides, predicting response to therapy and detecting complications are also a new clinical application aspect of MRTA in MI.

In addition to the above future developments that MRTA has potential in risk stratification and prognosis prediction in MI, the combined application with MRTA also has a certain prospect. First, except for the cine, mapping, and LGE, applying MRTA to other CMR quantitative techniques, such as strain, diffusion tensor imaging (DTI), perfusion-weighted imaging (PWI), and intravoxel incoherent motion (IVIM), can enrich the clinical research methods of MI. Second, MRTA combined with other imaging techniques, such as ECG, echocardiography, and myocardial radionuclide tomographic imaging may improve early MI diagnosis and prognosis. Third, cTn is the most diagnostic biochemical marker for MI, the combination of MRTA and cTn will increase the understanding of the connection between texture change and progression of MI. Fourth, the combination of radiomics characteristics and genomic data is described as radiogenomics (11). Gene regulation plays an important role in the occurrence of MI and cardiac remodeling after MI (72, 73). In the future, combinations of TA features and genomic data may have potential clinical application value in MI.

## Conclusion

At present, the inferential diagnosis of MI is based on the combination of biochemical markers, ischemic symptoms, or ECG changes. We are entering an era of combined imaging and clinical assessment in disease detection and diagnosis. MRTA can detect the invisible signal changes, thus, provides an additional non-Invasive method to establish the diagnosis, determine the age of infarct, and stratify risk and predict prognosis in MI. Although its current application for MI imaging faces some challenges, MRTA shows good clinical application prospects in MI.

## Author Contributions

FP conducted the reference analysis and wrote the manuscript. LGG and HZ contributed to the topic conception, manuscript revision, and decision to submit for publication and are the co-corresponding authors. The remaining authors contributed to the reference collection and helped in the revision of the manuscript. All authors contributed to the article and approved the submitted version.

## Funding

This study was supported by the National Natural Science Foundation of China (Grand No. 81860316).

## Conflict of Interest

The authors declare that the research was conducted in the absence of any commercial or financial relationships that could be construed as a potential conflict of interest.

## Publisher's Note

All claims expressed in this article are solely those of the authors and do not necessarily represent those of their affiliated organizations, or those of the publisher, the editors and the reviewers. Any product that may be evaluated in this article, or claim that may be made by its manufacturer, is not guaranteed or endorsed by the publisher.
